# Association between Resting Energy Expenditure and Heat Pattern in Traditional Medicine

**DOI:** 10.1155/2020/4093731

**Published:** 2020-03-25

**Authors:** Sujeong Mun, Kwang-Ho Bae, Kihyun Park, Siwoo Lee

**Affiliations:** ^1^Future Medicine Division, Korea Institute of Oriental Medicine, Daejeon, Republic of Korea; ^2^Clinical Medicine Division, Korea Institute of Oriental Medicine, Daejeon, Republic of Korea

## Abstract

Many symptoms of heat pattern (HP) in traditional medicine are related to thermoregulation; however, research on the association between energy expenditure characteristics and HP is limited. We aimed to study the association between HP and resting energy expenditure (REE). A total of 109 participants were divided into the HP and non-HP groups based on a self-administered questionnaire and their REE was measured using an indirect calorimeter. Multiple logistic regression models were used to calculate the odds ratio (OR) of HP according to the level of REE. No significant differences in age, anthropometric, and body composition characteristics were observed between the HP and the non-HP groups. The likelihood of having an HP significantly increased with higher REE after adjustment for fat-free mass (OR 1.22 [95% CI 1.03–1.46]) and also after additional adjustment for sex and age (OR 1.21 [95% CI 1.01–1.46]). These results suggest that increased REE could be a biological characteristic of HP. Future studies are necessary to investigate the underlying mechanisms associated with the differing energy expenditure in HP.

## 1. Introduction

Traditional medicine (TM) pattern is included in the eleventh edition of the International Classification of Diseases (ICD-11), which adopted the terminology from traditional medicine and can be found in one of its chapters along with terminologies for conventional medicine [1]. Pattern identification, which is sometimes also called as syndrome differentiation or Bian Zheng, has been primarily used to guide medical interventions in TM. Heat pattern (HP) is one of the major components of TM patterns [2, 3]. According to a survey in Korea, around 85% of TM doctors reported that they took the heat and cold patterns into consideration when prescribing herbal treatment. They also mentioned that consideration of heat and cold patterns was effective in a wide range of diseases including menopausal disorders, chronic rhinitis, dyspepsia, hwa-byung, diarrhea, dysmenorrhea, headache, inflammation in the digestive tract, coldness in the hands and feet, and atopic dermatitis [4]. It has also been published that identification of HP in rheumatoid arthritis could improve the successful treatment outcome rate when used with conventional biomedicine-based treatment [5, 6].

The symptomology of HP includes an aversion to heat, preference for coolness, thirst for cold drinks, redness of the face and eyes, hot limbs, constipation, and reduced output of urine, which is yellow. Many symptoms of HP are related to thermoregulation and/or thermal perception. In the modern study of thermoregulation, energy expenditure characteristics have been shown to have a significant impact on an individual's response to ambient temperature changes [7]. However, research on the association of energy expenditure characteristics with HP is limited. One study reported that resting energy expenditure (REE) of individuals with a warm sensation in the limbs and abdomen is greater than in those with a cold sensation; however, the differences disappeared after adjustment for age, sex, and body mass index (BMI) [8]. Another study reported that REE was positively associated with HP scores, which was defined using factor analysis of common symptoms used in the basic examination; however, the association was not significant after adjustment for sex and age [9]. To date, no study has focused on HP along with its comprehensive symptoms and considered body composition characteristics as confounders in the analysis of the association between HP and REE.

Therefore, in this study, we evaluated HP with seven comparatively comprehensive symptoms using a reliable questionnaire. REE was indirectly measured using a calorimeter and was further adjusted for possible confounders to elucidate a direct relationship between REE and HP.

## 2. Materials and Methods

### 2.1. Participants

This cross-sectional study was conducted between December 2014 and March 2015. The HP, body composition, and resting energy expenditure data were derived from the Korean Medicine Data Center of the Korea Institute of Oriental Medicine [10]. The institutional review board of the hospital approved the study protocol (KOMCIRB-140923-HR-007, KHNMCIH 2014-09-010), and informed consent was obtained from all participants prior to inclusion in the study.

Healthy individuals between the ages of 35 and 44 years with a BMI between 18.5 and 25 kg/m^2^ participated in the study. Individuals were excluded if they had undergone treatment for any internal, neurological, or psychological disorder within 6 months prior to the study; or had cardiovascular, cerebrovascular, or respiratory disorders, had sleeping problems (an average sleeping time shorter than 7 or longer than 8 hours, or a Pittsburgh Sleep Quality Index score of >5), fatigue (a score of ≥19 on the Chalder Fatigue Scale), and pain (a pain score of ≥40 mm on the visual analog scale). Individuals who were currently smoking, pregnant, breastfeeding, menstruating, participating in other clinical trials, experiencing tremors of the hands, or had arrhythmia were also excluded. A total of 130 healthy adults participated in the study. Among them, 109 participants who successfully maintained a steady state during the REE measurements were finally included in the analysis.

### 2.2. Data Collection

The symptoms of HP were evaluated using a self-administered questionnaire. The development of the questionnaire and its reliability has been reported previously [11]. Briefly, the relevant symptoms of HP were screened through a review of previously published questionnaires for HP and were selected through a discussion of TM experts. Twelve symptoms were originally included; however, only seven symptoms having the highest interitem consistency were included in the final version of the questionnaire (Table 1). Cronbach's coefficient was reported to be 0.83, and the intraclass correlation coefficient examining the test-retest reliability of the questionnaire was reported to be 0.78. Questions were evaluated on a 4-point scale as follows: 1 = strongly disagree; 2 = disagree; 3 = agree; and 4 = strongly agree. The heat pattern score (HPS) was calculated as the sum of the seven answers (range: 7–28). Respondents whose HPS were above the median value within each sex were categorized as HP and the others as a nonheat pattern (NHP).

Body height was measured with a digital stadiometer (BSM370, InBody, Seoul, South Korea). Weight, FFM, and FM were measured using a multifrequency bioelectrical impedance analysis (Inbody720, InBody, Seoul, South Korea). The BMI was calculated as weight in kilograms divided by the square of height in meters.

REE was measured using a breath-by-breath gas exchange analysis with an indirect calorimeter (Quark CPET, Cosmed, Italy). Participants were advised to fast for at least 8 hours prior to visiting the laboratory in the morning. They were directed to rest for 20 min in the supine position before measurement. Subsequently, the participants were placed under the canopy of the calorimeter and were directed to remain awake, but not speak; measurements were taken for 16 min. VO_2_ and VCO_2_ were averaged at 30 s intervals. The measurements from the first 5 min were discarded to minimize the effect of an unsteady state of the participants. Measurements from the remaining 11 min were visually inspected to detect a steady-state. REE was calculated with the Weir equation [12].

### 2.3. Statistical Analyses

Sample characteristics were presented as the mean and standard deviation. Age, anthropometrics, body composition, and REE of HP and NHP within each sex were compared by an independent *t*-test for those with normal distribution or by the Mann–Whitney *U* test for those with nonnormal distribution, while the Shapiro–Wilk test was used to assess the normality of data. To estimate the magnitude of the association between REE and HP, multiple logistic regression models were used to calculate the odds ratios (ORs) and 95% confidence intervals (95% CI) of HP. FFM, sex, and age were included in the adjusted models. The unit of REE in the multiple logistic regression models was 100 kcal/day so that the ORs can be interpreted as the change in OR of HP with each 100 kcal/day changes in REE. A *P* value of less than 0.05 was considered a statistically significant difference. All statistical analyses were performed using R (the R Foundation for Statistical Computing, Version 3.6.0).

## 3. Results

The characteristics of participants are presented in Table 2. The mean age of men and women were 37.9 and 39.3 years, respectively. In both men and women, there were no significant differences between the HP and NHP groups in terms of age, anthropometric, and body composition characteristics. REE of the HP group was higher than that of the NHP group; however, the statistical significance was marginal (*P*=0.077 in men; *P*=0.063 in women).

The ORs of HP according to REE are shown in Table 3. The likelihood of having an HP significantly increased with a higher REE in the adjusted models; the OR was 1.22 [95% CI 1.03–1.46] when FFM was adjusted (Model 2), and 1.21 [95% CI 1.01–1.46] when sex and age were additionally adjusted (Model 3). When the analysis was restricted to men, the association was not significant. In women, the OR of having HP significantly increased with higher REE in the adjusted models (OR 1.35 [95% CI 1.02–1.86] in Model 2; OR 1.35 [95% CI 1.02–1.86] in Model 3).

## 4. Discussion

This study examined the association between the level of energy expenditure and HP. Increased REE was found to be associated with a significantly increased likelihood of HP. These results suggest that increased REE could be one of the distinguished biological characteristics of HP.

REE typically accounts for the largest portion of total energy needs (approximately 65–70%) and is reported to predict energy intake by controlling the hunger signal [13]. Therefore, determining the REE is essential for the nutrition of the general population, athletes, and severely ill patients. Altered REE is reported to be related to obesity, diabetes mellitus, metabolic syndrome, multimorbidity, and mortality [14–18].

Measuring REE with an indirect calorimeter is deemed to be the gold-standard method; however, this method is not always feasible in clinical settings. Therefore, various equations for estimation have been developed based on a subset of comparatively easily measurable covariates such as age, sex, height, weight, or body composition [19, 20]. In the present analysis, we used total body FFM, an energetically heterogeneous compartment with organs and tissues having different metabolic rates [21]; therefore, the possibility that differences in the composition of FFM contributed to differences in REE between the HP and the NHP groups cannot be excluded. In our study, however, the association of REE with HP was significant even after normalization for FFM and further adjustment for sex and age. This suggests that other determinant factors of REE, such as sympathetic nervous system activity and endocrine status, might contribute to increased energy expenditure in the HP group.

In this study, the association between REE and HP was significant when all participants were included; however, when the analysis was confined to each sex, the association was only significant in women. It has been reported that thermoregulatory responses differ according to sex. Women have a greater ratio of body surface to body mass, more subcutaneous fat content, lower exercise capacity, difference in control of cutaneous blood flow, and reduced sweating response to heat load when compared to men [22, 23]. Moreover, women are more sensitive to ambient temperatures, which could make them well aware of HP-related symptoms (aversion to heat, preference for coolness, and a warm sensation in the body) when compared to men [24]. Therefore, the differences in these characteristics could lead to a difference in the magnitude of association of REE levels with the presence of HP between the sexes, although the detailed mechanism behind this is unclear.

The two previous studies that evaluated the association of REE with HP symptoms showed similar results to ours. However, unlike in our study, the significance disappeared when adjusted for other confounders [8, 9]. In those studies, HP was defined by only a few HP-related symptoms or was extracted using a factor analysis of common symptoms that were used in the basic examination. In our study, we included comparatively comprehensive HP-related symptoms to differentiate between HP and NHP using a questionnaire which showed a good-to-excellent internal consistency and test-retest reliability [11]. The differences in the way of defining the HP are assumed to be the main reason for the differences in results between our study and the previous studies.

Some limitations should be considered when interpreting the results. First, the main purpose of our analysis was to evaluate whether the level of REE was associated with HP in healthy people; therefore, we purposely did not focus on a particular disease, which may limit the generalization of our results to a specific disease. Second, a consensus on the method of evaluating HP has not yet been reached among numerous clinicians and researchers. Although we have used the HP questionnaire that has previously been shown to be reliable, if the evaluation tool for HP is changed, the energy expenditure characteristics of HP could be different from ours. Third, the menstrual cycle of women was not considered in the present analysis. As the menstrual cycle is reported to be a significant contributor to variation in REE [25], future studies should include the menstrual cycle to further the association of REE with HP. Fourth, the REE steady-state was detected by visually inspecting the measurement, rather than by using quantitative standards. Future studies are recommended to use VO_2_ and VCO_2_ coefficients of variation to calculate the steady-state REE.

## 5. Conclusions

The likelihood of having HP increased with higher REE. These results suggest that increased REE could be one of the distinguished biological characteristics of HP. Differed REE might influence the energy balance of an individual, and the risk of obesity and other metabolic diseases could be different between the HP and NHP groups. Furthermore, underlying mechanisms that lead to the differences in REE of HP might cause differences in the pathophysiology of a certain disease. Future studies are necessary to investigate the underlying mechanisms associated with differed energy expenditure in HP.

## Figures and Tables

**Table 1:** Symptoms constituting the heat pattern questionnaire.

1. I usually have an aversion to heat
2. I usually prefer cool or cold
3. I usually have a warm sensation in the body or feel hot
4. I usually feel a hot or burning sensation in the body
5. My face or eyes are usually reddish
6. I usually drink cold/cool water
7. My breath is usually hot

**Table 2:** Characteristics of the study participants according to sex and the presence of heat pattern.

	Men				Women			
	HP (*N* = 22)	NHP (*N* = 26)	Total (*N* = 48)	*P*	HP (*N* = 23)	NHP (*N* = 38)	Total (*N* = 61)	*P*
Age (years)	37.8 ± 2.7	38.0 ± 2.9	37.9 ± 2.8	0.975	38.9 ± 2.9	39.6 ± 3.2	39.3 ± 3.1	0.415
Height (cm)	173.3 ± 5.7	173.1 ± 5.7	173.2 ± 5.6	0.836	160.9 ± 5.4	160.4 ± 5.9	160.6 ± 5.7	0.711
Weight (kg)	70.8 ± 7.6	69.5 ± 6.9	70.1 ± 7.1	0.520	57.2 ± 6.2	56.5 ± 7.8	56.8 ± 7.2	0.697
BMI (kg/m^2^)	23.6 ± 1.9	23.2 ± 2.0	23.4 ± 2.0	0.508	22.1 ± 1.9	21.9 ± 2.4	22.0 ± 2.2	0.682
FFM (kg)	55.3 ± 5.5	55.2 ± 5.8	55.3 ± 5.6	0.957	39.7 ± 4.5	40.1 ± 4.5	40.0 ± 4.4	0.735
FM (kg)	15.5 ± 4.8	14.3 ± 2.8	14.9 ± 3.9	0.290	17.5 ± 3.7	16.3 ± 4.8	16.8 ± 4.4	0.277
REE (kcal/day)	2005.3 ± 301.3	1859.7 ± 243.5	1926.2 ± 278.1	0.077	1499.8 ± 222.5	1394.2 ± 199.1	1432.9 ± 212.4	0.063

HP, heat pattern; NHP, nonheat pattern; REE, resting energy expenditure; BMI, body mass index; FFM, fat-free mass; FM, fat mass. Values are represented as the mean ± standard deviation. Differences between NHP and HP were assessed by an independent *T*-test or the Mann–Whitney *U* test.

**Table 3:** Odds ratio (95% confidence interval) of the heat pattern according to resting energy expenditure.

		Model 1		Model 2		Model 3	
		OR (CI)	*P*	OR (CI)	*P*	OR (CI)	*P*
All	REE^*∗*^	1.11 (1.00–1.25)	0.058	1.22 (1.03–1.46)	**0.029**	1.21 (1.01–1.46)	**0.039**
	FFM	—		0.96 (0.89–1.02)	0.194	0.96 (0.88–1.05)	0.379
	Sex: female	—		—		1.08 (0.23–5.20)	0.922
	Age	—		—		0.96 (0.83–1.10)	0.538

Men	REE^*∗*^	1.10 (0.90–1.36)	0.367	1.13 (0.89–1.44)	0.317	1.13 (0.89–1.44)	0.325
	FFM	—		0.97 (0.86–1.10)	0.658	0.98 (0.86–1.10)	0.691
	Age	—		—		0.98 (0.79–1.21)	0.871

Women	REE^*∗*^	1.29 (0.99–1.73)	0.068	1.35 (1.02–1.86)	**0.047**	1.35 (1.02–1.86)	**0.050**
	FFM	—		0.94 (0.82–1.07)	0.340	0.95 (0.83–1.08)	0.448
	Age	—		—		0.94 (0.78–1.12)	0.494

Model 1, unadjusted; Model 2, adjusted for FFM; Model 3, adjusted for FFM, sex, and age; CI, confidence interval; HP, heat pattern; OR, odds ratio; REE, resting energy expenditure. ^*∗*^The unit of REE is 100 kcal/day.

## Data Availability

The data used to support the findings of this study were supplied by the Korean Medicine Data Center of the Korea Institute of Oriental Medicine under license and so cannot be made freely available. Requests for access to these data should be made to the Korean Medicine Data Center (http://kdc.kiom.re.kr/html/).
